# Plagiarism detection – quality management tool for all scientific journals

**DOI:** 10.3325/cmj.2012.53.1

**Published:** 2012-02

**Authors:** Ksenija Baždarić

**Affiliations:** Department of Medical Informatics, Rijeka University School of Medicine, Rijeka, Croatia

Plagiarism detection software has considerably affected the quality of scientific publishing. No longer is plagiarism detection done by chance or is the sole responsibility of the reviewer and reader (1). The *Croatian Medical Journal* (*CMJ*) appointed Research Integrity Editor in 2001, which paved the way for the introduction of computer detection of plagiarism (2,3).

The story began when Mladen Petrovečki and Lidija Bilić-Zulle, members of the *CMJ* Editorial Board, came up with the idea to measure the prevalence of and attitudes toward plagiarism in the scientific community, as a follow-up to their investigation on plagiarism among students (4,5). Together with Matko Marušić and Ana Marušić, Editors-in-Chief, and Vedran Katavić, Research Integrity Editor, they developed a procedure for detecting and preventing plagiarism using plagiarism detection software, which later became a standard (1,6). The study of research integrity started in the early 2000s at the Rijeka University School of Medicine as part of two consecutive projects supported by the Ministry of Science, Technology, and Sports. Even outside our small scientific community, the projects were recognized as valuable and obtained a Committee on Publications Ethics (COPE) grant in 2010. Membership in the CrossRef association (*http://www.crossref.org/*) and the introduction of CrossCheck (*http://www.crossref.org/crosscheck/index.html*), a unique web-service for detecting plagiarism in scientific publications, marked the beginning of a new era at the *CMJ*.

In 2009, we started to systemically check all the submitted manuscripts. The plagiarism detection procedure consisted of automatic scanning of manuscripts using plagiarism detection software (eTBLAST and CrossCheck) and manual verification of manuscripts suspected of having been plagiarized (more than 10% text similarity). The criteria for plagiarism were set according to the prior investigations carried out by Bilić-Zulle et al (4,5) and Segal et al (7), and the definition of redundant publication used by the *British Medical Journal* (8). Manual verification (reading of both manuscripts) was done according to the COPE's flowcharts (9) and the *CMJ*'s Guidelines for Authors. Over two years, we detected 85 manuscripts (11%) containing plagiarized parts (8% true plagiarism and 3% self-plagiarism) (6).

CrossCheck is an excellent service for detecting plagiarism, which detected almost all plagiarized manuscripts in our study. eTBLAST was less informative, possibly because at the time of the investigation it only had the ability to compare the text with abstracts from the Medline database (today eTBLAST searches abstracts in Medline, Pub Med Central, Clinical Trials, Wikipedia, and other databases outside the field of medicine).

If a suspected case of copy/paste activity was found, the investigator wrote a plagiarism report to the Editorial Board to assist in deciding on the manuscript’s status. Editors mostly accepted the suggestions and in case of disagreement, the final decision lay with the Research Integrity Editor. Cases of blatant plagiarism were easy to deal with because of text similarity in all sections of the manuscript, while those with less text similarity were sometimes more complicated and COPE’s flowcharts were not sufficient to conclude whether the manuscript was plagiarized.

Special attention was paid to plagiarism in the Results section. Also, there was zero tolerance for plagiarism in the Discussion section. When manuscripts contained plagiarism in the Materials and Methods section or when the original article was not cited in follow-up investigations, accidentally or by ignorance, authors were given an opportunity to rewrite the text and publish their investigation. These examples once again show that it is of genuine importance for editors to become educators, ie, to teach authors about standards in publishing and research through continuing education (10).

We believe that the main reasons for plagiarizing were unawareness of research integrity policies, poor English proficiency, attitudes toward plagiarism, and cultural values (6,11-13). In Croatia, the situation could be further deteriorated by a new law on science, higher education, and universities that abolishes the Committee for Ethics in Science and Higher Education, the highest national body dealing with research integrity (14). Integrity issues and education of future scientists about the responsible research conduct will now be the task of Croatian universities and schools only. Also, since in the academic community there is a considerable pressure to publish and since English is not the first language in Croatia, some authors simply decide to “borrow” a portion of text from previous papers (11). In addition, it has been shown that in post-communist countries moral and cultural values and attitudes toward plagiarism are different from those in Western countries that have a longer tradition of high research integrity standards (15).

Plagiarism is not easy to define (16); there are still no criteria that are widely accepted by medical editors/journals as to what constitutes plagiarism. How much textual similarity raises the suspicion of plagiarism? Is it 5% or 10%, as stated by one source, or 100 words, as it was argued in the discussion of the COPE’s recent paper “How should editors respond to plagiarism?” (5-7,17)? Is there a difference between different types of plagiarism detection software? Plagiarism detection software offers valuable help in preventing plagiarism, but only if followed by manual verification (6). All manuscripts submitted to the journal should be checked and never rejected relying solely on the similarity report of plagiarism detection software (1,6). Therefore, medical editors are expected to reach a consensus on what constitutes plagiarism and make clear policies on how to deal with cases of plagiarism.

The *CMJ* was the first scientific journal in Croatia to begin checking all the submitted manuscripts for plagiarism (2009) and, to the best of my knowledge, together with the Chinese *Journal of Zhejiang University Science,* the only journal in the world that has systematically collected data on plagiarism in the submitted manuscripts. Furthermore, the *CMJ* is the first medical journal to publish the standard operating procedure for scanning submitted manuscripts (study protocol), as part of the journal’s “striving for excellence” policy (1,18).

Plagiarism detection software enables systematic detection and prevention of plagiarism, leading to fewer retractions. The results of our study were published (6) and we expect other medical journals to publish their results, not only a description of experiences. In order to reach high research integrity standards and journal quality, journals should perform systematic checking of all submitted manuscripts according to the widely accepted standards (protocols), as well as conduct ongoing education of authors.
